# Expression of Concern: Ramentaceone, a Naphthoquinone Derived from *Drosera sp*., Induces Apoptosis by Suppressing PI3K/Akt Signaling in Breast Cancer Cells

**DOI:** 10.1371/journal.pone.0211655

**Published:** 2019-01-29

**Authors:** The PLOS ONE Editors

After publication of this article [1], concerns were raised about results reported in Figs 2, 5, 7, and 8:

- Fig 2B: It was raised that the same image appears to be presented in the 0 and 0.5 μM panels for BT474, and that there are several areas of similarity in the 0.5 and 1 μM panels for SKBR3.
- Fig 5: Similarities were noted between several regions of the MCF-7 panels, and between several regions of the MDA-MB-231 panels.
- Similarities were noted between the BT474–10 panel of Fig 5 and the BT474 –Akt siRNA/5 μM ramentaceone panel of Fig 8B.
- Similarities were noted between the SKBR3–10 panel of Fig 5 and the SKBR3 –ctrl siRNA/5μM ramentaceone panel of Fig 8B.
- Similarities were noted between the background pixelation patterns of Bax, Bak, Bcl-2, and β-actin panels for the BT474 and SKBR3 experiments in Fig 7.
- Fig 8B: Similarities were noted between regions of three BT474 panels (ctrl siRNA non-treated, cntrl siRNA 5 μM ramentaceone, Akt siRNA non-treated), and between regions of three SKBR3 panels (cntrl siRNA non-treated, Akt siRNA non-treated, Akt siRNA 5 μM ramentaceone).

The *PLOS ONE* Editors have notified the University of Gdansk and Medical University of Gdansk of these concerns and issue this Expression of Concern so readers are aware of the issues whilst they are investigated.
